# Using a Development Platform with an STM32 Processor to Prototype an Inexpensive 4-DoF Delta Parallel Robot

**DOI:** 10.3390/s21237962

**Published:** 2021-11-29

**Authors:** Pawel Andrzej Laski, Mateusz Smykowski

**Affiliations:** Department of Automation and Robotics, Faculty of Mechatronics and Machine Design, Kielce University of Technology, Aleja Tysiaclecia Panstwa Polskiego 7, 25-314 Kielce, Poland; msmykowski@interia.pl

**Keywords:** STM32 processor, parallel robot, 4-DoF, rapid control prototyping

## Abstract

This article presents a construction prototype of a delta 4-DoF (Degree of Freedom) parallel robot. The structure of kinematic chain was described and the problem of inverse kinematic was formulated and solved. The author also proposed a concept of a control system. The dynamics of the control object were specified, a decision upon the controller and its settings was made, as well as simulation control studies of manipulator drive were conducted. The article contains a description of prepared applications and procedures as well as the research results of the manipulator.

## 1. Introduction

Recently, there has been a significant increase in construction of parallel robots [1,2,3,4,5,6,7,8]. Manipulators and parallel robots are more widely applied in industry. Devices of closed kinematic structure perform much better in many applications than typical industrial robots of serial kinematics [9,10]. Robots with closed kinematic chain are characterized by large rigidity, better positioning repeatability, and manipulation precision [6,11,12,13,14,15,16,17]. Robots also have better capacity, and might achieve better acceleration and end-effector velocity [18,19]. Due to their fast response and movement precision, parallel robots are mainly applied in pick-and-place applications, which require manipulation of transported goods in four axes. In a typical three-armed delta robot [20,21], the fourth degree of freedom is achieved due to the application of a telescope shaft with cardan joints which is responsible for end-effector rotation on a moveable platform (this solution is to be noticed in, e.g., ABB of Fanuc parallel robots) [22]. There are also parallel manipulators with four kinematic chains and of four degrees of freedom. An example thereof might be a parallel architecture Par4 suggested by engineers from LIRMM and Fatronik-Tecnalia foundation.

Par4 [23,24] is a parallel robot with four degrees of freedom that belongs to a group of manipulators with a special articulated construction of a moveable platform. It is composed of four identical kinematic chains, four drives symmetrically arranged on the drives’ basis (of rotary movement) in relation to the vertical axis of robot base system, and a symmetrical moveable articulated platform. The platform consists of four elements—two main parts joined with two connectors with the use of rotating joints. Such construction is in the form of a flat articulated quadrangle and provides a rotating movement along the vertical axis (the fourth degree of freedom) [25]. The constructional range of platform movement is only ±45°. Thus, in a prototype construction, the constructors of Par4 [25,26,27] robots applied a special multiplying mechanism which enables a full movement ±180°. Robots have a belt drive (1:4 position) with the first wheel rigidly fixed on the first part of the moveable platform, and the second one joined with the second main part of the articulated quadrangle of the platform with the use of a rotating joint. The moveable wheel is at the same time a robot disc where tools or grabs are fixed. Such solution does not require application of a telescope shaft, which is centrally located on the robot basis, in order to provide the fourth degree of freedom (rotation around a vertical axis). There are also other constructions of moveable articulated platforms of parallel robots of four degrees of freedom that are suggested by engineers from LIRMM. They include, e.g., H4, I4L, and I4R [28].

The first commercial version of a parallel robot Par4 was the model Quattro s650H manufactured by Adept Technology [29]. In 2005, this robot was patented by Adept, which is the only company that has an exclusive license to sell it [30]. The robots produced by Adept Technology (models s650H and s800H) are distinguished from parallel robots by their unique construction (Par4 parallel architecture) [31,32]. Adept company refers to parallel robots as delta robots of four degrees of freedom.

In Adept Quattro robot—in comparison to a typical mechanism of delta kinematic structure—four identical arms and a moveable articulated platform with an innovative solution of the movement of the operating end were applied. Connection of four kinematic chains increases total rigidity of the robot, and thus, decreases the load of particular elements thereof, and allows to achieve high operation velocity and acceleration, transport heavy loads, and achieves higher efficiency in total robot operating space. The moveable articulated platform converts the movement of four drives into a movement in Cartesian coordinate system and processes the rotation angle of the robot tool. The tool rotation is performed by a special gear mechanism (belt or toothed) or directly, in case of applications requiring greater rotational force, but lower rotational angle. Depending on the performance for both types of robots (s650H and s800H), the platform might rotate within the following ranges: ±46.25°, ±92.5°, or ±185° (it is possible to order a robot with a rigid platform, with no rotation) [33,34].

Robots Quattro s650H and s800H manufactured by Adept Technology are applied first of all in applications requiring great velocity (packing, transport and assembly, mainly with the use of vision systems). Robots Quattro are characterized by great range of operation (respectively, ø1300 × 500 and ø1600 × 500), large repeatability, (±0.10 and ±0.15 mm), short cycles, and large maximum capacity (4 and 6 kg) [35].

## 2. Solid Model of Delta 4-Dof Manipulator

**Figure 1:**
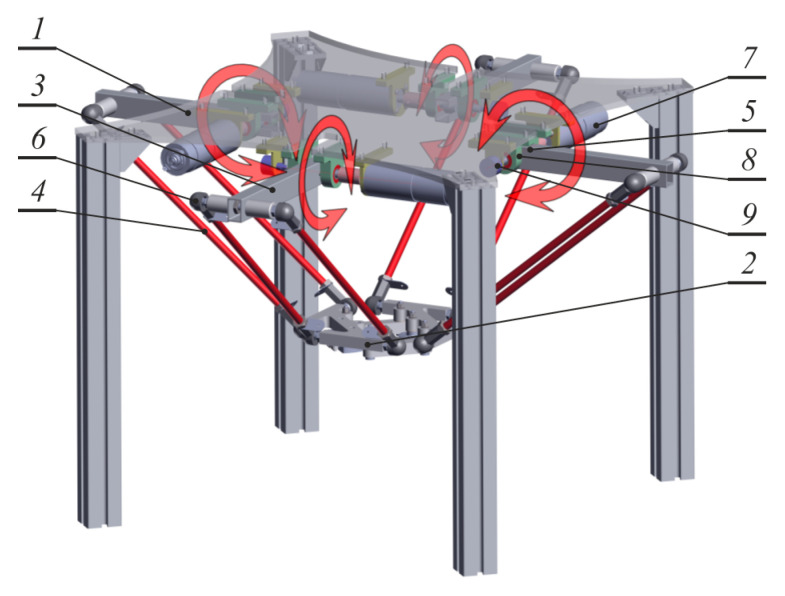
Solid model of delta 4-DoF parallel manipulator. 1—basis; 2—operating platform; 3—active arm; 4—passive arms; 5—rotating joint; 6—spherical joint; 7—drive (DC motor); 8—bearing bracket; 9—sensor of angular position.

The manipulator was designed on the basis of the construction of parallel robots of four degrees of freedom produced by Adept Technology. Its solid model, which was prepared with the use of 3D CAD, is presented in Figure 1. The robot consists of an immoveable basis, moveable articulated platform, and four identical kinematic chains connecting them.

The immoveable basis (load-bearing frame) of the robot is based on the plate to which four aluminum profiles were attached as columns supporting the robot construction. In order to improve frame rigidity and prevent undesirable change of profiles’ direction, stiffening angle brackets between the columns and basis of the robot were applied. The robot is symmetrical in relation to the basis center. Robot mechanisms are arranged on the basis of a plan of a square (evenly every 90°).

Each kinematic chain consists of active and passive drive elements. A drive arm (active) is an aluminum closed profile of a square cross-section, which is mounted to the shift on one side, and its second end is pinned to passive elements of the shaft with the use of spacer elements. The shaft is mounted in the rotating joint of one degree of freedom that connects the active arm with the robot basis. The rotation axis of the shaft is connected with the sensor of angular position of the robot arm.

In order to process the movement of the drive arm to the moveable operating platform, two glass-fiber rods (of identical length and diameter) ended with spherical joints were applied.

Four motors of direct current with an integrated planetary gearbox with the ratio 264 constitute the drive of the manipulator. Drive transfer from the motors to the active robot elements takes place with the use of flexible backlash-free couplings connecting the input shaft of the motor gearbox with the shaft of the rotating arm.

**Figure 2:**
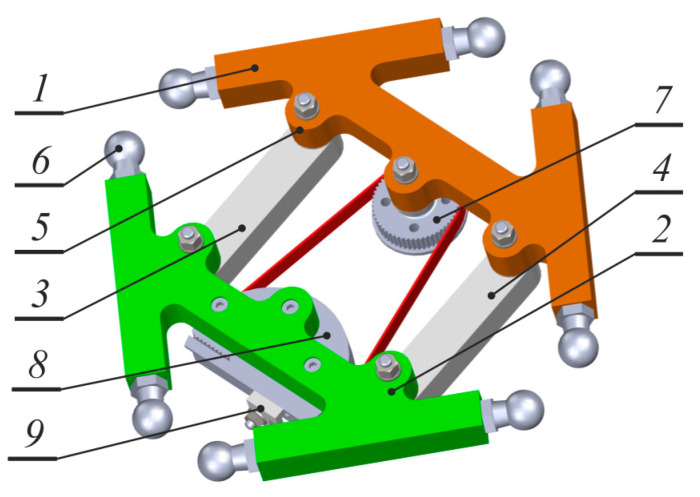
Operating platform of the designed manipulator. 1, 2—main elements; 3, 4—connectors; 5—rotary joint; 6—ball pin; 7—toothed wheel; 8—immoveable “wheel”; 9—tensioning belt.

Figure 2 presents a general view of the operating platform. The operating platform consists of four elements connected with the use of rotating joints: two main parts (1,2) and two connectors (3,4). Such a construction of the operating platform provides an additional fourth degree of freedom—platform rotation around the vertical axis of the robot. Due to the mechanical restrictions of the platform, a special mechanism which allows full rotation ±90° (1:2 location) was applied. This mechanism has a structure of a belt gearbox. A belt gearbox consists of three parts: an element in the form of an incomplete wheel (along which a toothed wheel rotates) which is firmly fixed to one part of the moveable platform; cogwheel connected with the second part of the quadrangle of the operating element and the toothed belt with the use of a rotating joint. The immoveable wheel has an outlet opening in the form of a belt outline which allows to be mounted with one end. The immoveable wheel is equipped as well with a tensioner, which, due to a profile opening, makes shifting it possible in order to achieve proper tension of the toothed belt.

## 3. Kinematic Analysis of Delta 4-DoF Manipulator

### 3.1. Description of Kinematics of Delta 4-DoF Manipulator

**Figure 3:**
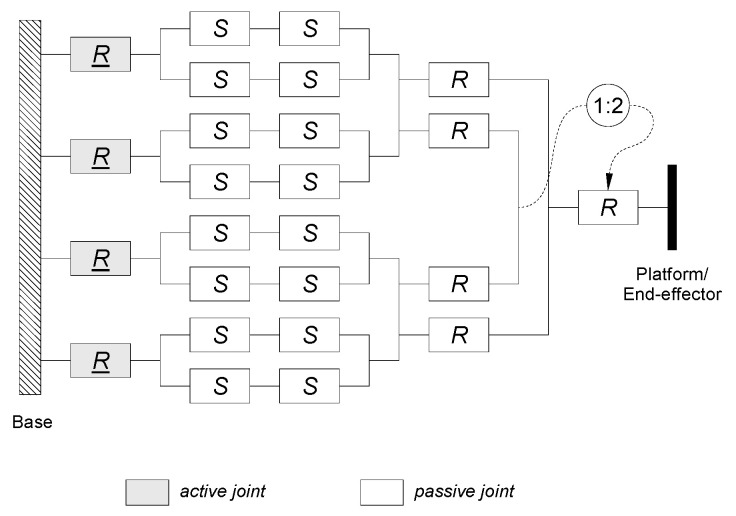
Kinematic diagram of the mechanism of delta 4-DoF robot.

The project of robot mechanical construction is based on a parallel *delta* 4-DoF structure. The manipulator consists of a basis placed on a load-bearing structure, moveable articulated platform, and four identical two-element arms. Each arm creates a kinematic chain R-(SS)_2_, which includes active rotary connections R and spherical joints S, which form the ends of passive elements—quadrangles transferring motion to the moveable platform. The moveable platform has a structure of an articulated quadrangle (it has four elements connected by rotary joints R). Figure 3 shows a diagram of the kinematic structure of the designed manipulator.

### 3.2. Inverse Kinematics of Delta 4-DoF Manipulator

**Figure 4:**
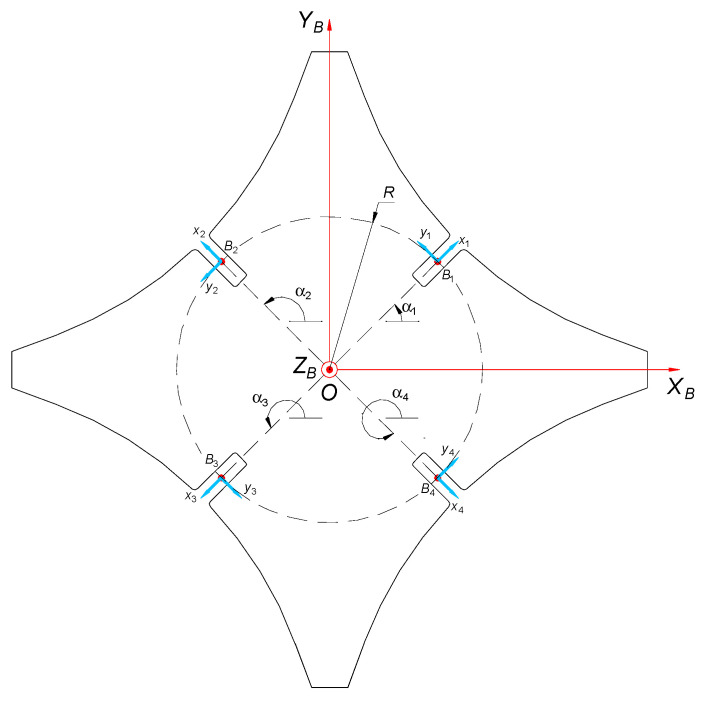
Overview of the immoveable manipulator basis with indicated characteristic geometrical points.

The solution of inverse kinematic for the designed *delta* 4-DoF manipulator consists of determining configuration variables *q_i_* (*i* = 1, 2, 3, 4) as functions of position *^O^P* = [*X Y Z*]^T^ and orientation *θ* of the operating element and already known geometric parameters of the robot. A global (Cartesian) coordinate system *OX_B_Y_B_Z_B_*, as shown in Figure 4, is related to the center of the robot basis (point *O*).

The manipulator has four kinematic chains arranged in accordance with Figure 4. The angular placement of particular robot arms is the following:

$$\alpha_{1}=45^{{}^{\circ}}\;\;\;\;\;\;\;\;\alpha_{2}=135^{{}^{\circ}}\;\;\;\;\;\;\;\;\alpha_{3}=225^{{}^{\circ}}\;\;\;\;\;\;\;\;\alpha_{4}=315^{{}^{\circ}}$$

The beginning of each arm is in point *B_i_* that belongs to a circle with its middle in point *O* and radius *R*. The coordinates of point *B_i_* for *i*-arm are:

(1) BiO=[RcosαiRsinαiBiz](i=1, 2, 3, 4)

**Figure 5:**
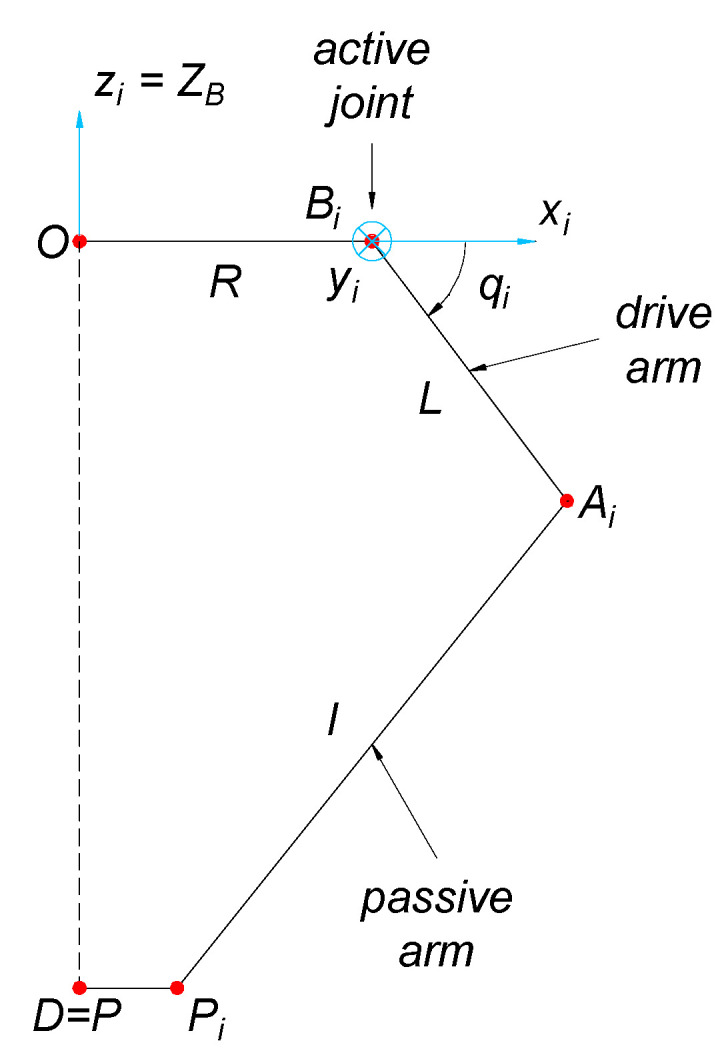
Overview of one arm with indicated characteristic dimensions.

Figure 5 presents the manipulator projection on a plane that is parallel to one arm with indicated characteristic geometric dimensions that result from the manipulator’s mechanical construction. For each arm in point *B_i_*, a new reference system *B_i_x_i_y_i_z_i_* was assumed. Axis *y_i_* overlaps with the rotation axis of the rotating joint of the active arm under consideration, and the axis *z_i_* is parallel to axis *Z_B_*. The length of the active element is indicated by *L* and is placed between the points *B_i_* and *A_i_*. The passive arm is of length *l* that is measured between points *A_i_* and *P_i_*. The coordinates of point *A_i_* (which is the center of the segment connecting the centers of rotating joints S on *i*-arm) in the system *B_i_x_i_y_i_z_i_* might be easily determined as the functions of sought configuration variables *q_i_* and dimension *L*:

(2) AiBi=[Lcosqi0−Lsinqi](i=1, 2, 3, 4)

However, the coordinates of vector *A_i_* expressed in reference system of the basis are:

(3) AiO=[LcosqicosαiLcosqisinαi−Lsinqi](i=1, 2, 3, 4)

**Figure 6:**
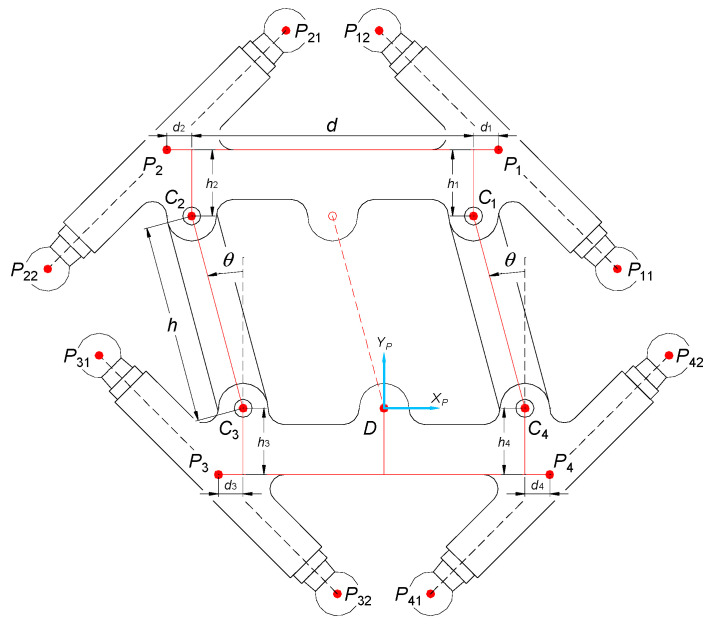
Kinematic relations of the moveable platform.

Figure 6 presents the overview of a moveable platform with its most important geometric parameters. The system of coordinates, where the coordinates of joints that belong to the moveable platform are defined, is related to the platform in point *D* (which constitutes its “center of the local reference frame”). The following geometric dimensions are related to the operating element:

*d*—length of the main element of the moveable platform (along the axis *x*),
*h*—length of the connectors of the articulated quadrangle of the moveable platform (measured between points *C*_1_ and *C*_4_ and *C*_2_ and *C*_3_),
*d_i_* and *h_i_*—distances between points *C_i_* and *P_i_* (along the axes *x* and *y*, respectively),
θ—rotary angle of the articulated quadrangle of the moveable platform that results from its constructional limitations (±45°, without the mechanism increasing the range of movement).

Points *C_i_* are centers of the rotating joints connecting all elements of the articulated quadrangle of the moveable platform. Points *P_i_*_1_ and *P_i_*_2_ are the centers of spherical joints connected with the moveable platform. The location of points *P_i_* (which are the centers of the segments connecting points *P_i_*_1_ and *P_i_*_2_) might be expressed as the functions of orientation of the final end-effector *θ* and some geometric constants (*d*, *d_i_*, *h*, *h_i_*):

(4) P1D=[−hsinθ+d2+d1hcosθ+h1P1z]

(5) P2D=[−hsinθ−d2−d2hcosθ+h2P2z]

(6) P3D=[−d2−d3−h3P3z]

(7) P4D=[d2+d4−h4P4z]

On the basis of the robot geometry (Figure 5) it was assumed that:

(8) BiO+AiO+liO=PO+PiD (i=1, 2, 3, 4)

By transforming the dependency (8) due to *l_i_* it was achieved:

(9) liO=PO+PiD−BiO−AiO (i=1, 2, 3, 4)

By substituting all required values to (9), the coordinates of vectors *l_i_* related to the *i*-arm of the robot were determined:

(10) l1O=[X+d2+d1−Rcosα1−hsinθ−Lcosα1cosq1Y+h1−Rsinα1+hcosθ−Lsinα1cosq1Z−B1z+P1z+Lsinq1]

(11) l2O=[X−d2−d2−Rcosα2−hsinθ−Lcosα2cosq2Y+h2−Rsinα2+hcosθ−Lsinα2cosq2Z−B2z+P2z+Lsinq2]

(12) l3O=[X−d2−d3−Rcosα3−Lcosα3cosq3Y−h3−Rsinα3−Lsinα3cosq3Z−B3z+P3z+Lsinq3]

(13) l4O=[X+d2+d4−Rcosα4−Lcosα4cosq4Y−h4−Rsinα4−Lsinα4cosq4Z−B4z+P4z+Lsinq4]

The kinematic relation connecting the given point *^O^P* = [*X Y Z*]^T^, which the operating end is to produce, with the sought configuration coordinates *q_i_* of the active joints is determined in the following way:

(14) l2=‖liO‖2=lix2+liy2+liz2 (i=1, 2, 3, 4)

After placing, respectively, (10), (11), (12), and (13) to (14), and then raising to the power and comparing, the dependency (14) was reduced to the system of four independent Equation (15). In order to implement and solve equations in the Matlab/Simulink software for the STM32F4 development platform, geometrical transformations and substitutions were made to Formula (14) and transformed to the form in Formula (15).

(15) $$E_{i}\sin q_{i}+F_{i}\cos q_{i}+G_{i}=0\;\left(i=1,\;2,\;3,\;4\right)$$

where: *E_i_*, *F_i_*, and *G_i_* are functions of the robot geometry. For the first arm they equal:

$$E_{1}=2LZ-2LB_{1z}+2LP_{1z}$$

(16) $$\begin{array}{cc} F_{1} & =2LR-2LX\cos\alpha_{1}-Ld\cos\alpha_{1}-2Ld_{1}\cos\alpha_{1}-2LY\sin\alpha_{1}-2Lh_{1}\sin\alpha_{1}+2Lh\cos\alpha_{1}\sin\theta \\ & -2Lh\sin\alpha_{1}\cos\theta \end{array}$$

$$\begin{array}{cc} G_{1} & =Xd+2Xd_{1}-2ZB_{1z}+2Yh_{1}+dd_{1}+2ZP_{1z}-2B_{1z}P_{1z}+L^{2}+R^{2}+X^{2}+Y^{2}+Z^{2}+B_{1z}^{2}+\frac{d^{2}}{4}+d_{1}^{2}\\ & +h^{2}+h_{1}^{2}-l^{2}+P_{1z}^{2}-2Xh\sin\theta +2hh_{1}\cos\theta -dh\sin\theta -2d_{1}h\sin\theta -2RX\cos\alpha_{1}-Rd\cos\alpha_{1}\\ & -2Rd_{1}\cos\alpha_{1}-2RY\sin\alpha_{1}-2Rh_{1}\sin\alpha_{1}+2Yh\cos\theta +2Rh\cos\alpha_{1}\sin\theta -2Rh\sin\alpha_{1}\cos\theta \end{array}$$

By analogy for the second, third, and the fourth arm:

$$E_{2}=2LZ-2LB_{2z}+2LP_{2z}$$

(17) $$\begin{array}{cc} F_{2} & =2LR-2LX\cos\alpha_{2}+Ld\cos\alpha_{2}+2Ld_{2}\cos\alpha_{2}-2LY\sin\alpha_{2}-2Lh_{2}\sin\alpha_{2}+2Lh\cos\alpha_{2}\sin\theta \\ & -2Lh\sin\alpha_{2}\cos\theta \end{array}$$

$$\begin{array}{ll} G_{2} & =2Yh_{2}-2Xd_{2}-2ZB_{2z}-Xd+dd_{2}+2ZP_{2z}-2B_{2z}P_{2z}+L^{2}+R^{2}+X^{2}+Y^{2}+Z^{2}+B_{2z}^{2}+\frac{d^{2}}{4}+d_{2}^{2}\\ & +h^{2}+h_{2}^{2}-l^{2}+P_{2z}^{2}-2Xh\sin\theta +2hh_{2}\cos\theta +dh\sin\theta +2d_{2}h\sin\theta -2RX\cos\alpha_{2}+Rd\cos\alpha_{2}\\ & +2Rd_{2}\cos\alpha_{2}-2RY\sin\alpha_{2}-2Rh_{2}\sin\alpha_{2}+2Yh\cos\theta +2Rh\cos\alpha_{2}\sin\theta -2Rh\sin\alpha_{2}\cos\theta \end{array}$$

$$E_{3}=2LZ-2LB_{3z}+2LP_{3z}$$

(18) $$F_{3}=2LR-2LX\cos\alpha_{3}+Ld\cos\alpha_{3}-2Ld_{3}\cos\alpha_{3}-2LY\sin\alpha_{3}+2Lh_{3}\sin\alpha_{3}$$

$$\begin{array}{ll} G_{3} & =L^{2}+R^{2}-2RX\cos\alpha_{3}-2RY\sin\alpha_{3}+Rd\cos\alpha_{3}+2Rd_{3}\cos\alpha_{3}+2Rh_{3}\sin\alpha_{3}+X^{2}-Xd-2Xd_{3}\\ & +Y^{2}-2Yh_{3}+Z^{2}-2ZB_{3z}+2ZP_{3z}+B_{3z}^{2}-2B_{3z}P_{3z}+\frac{d^{2}}{4}+dd_{3}+d_{3}^{2}+h_{3}^{2}-l^{2}+P_{3z}^{2} \end{array}$$

$$E_{4}=2LZ-2LB_{4z}+2LP_{4z}$$

(19) $$F_{4}=2LR-2LX\cos\alpha_{4}-Ld\cos\alpha_{4}-2Ld_{4}\cos\alpha_{4}-2LY\sin\alpha_{4}+2Lh_{4}\sin\alpha_{4}$$

$$\begin{array}{ll} G_{4} & =L^{2}+R^{2}-2RX\cos\alpha_{4}-2RY\sin\alpha_{4}-Rd\cos\alpha_{4}-2Rd_{4}\cos\alpha_{4}+2Rh_{4}\sin\alpha_{4}+X^{2}+Xd+2Xd_{4}\\ & +Y^{2}-2Yh_{4}+Z^{2}-2ZB_{4z}+2ZP_{4z}+B_{4z}^{2}-2B_{4z}P_{4z}+\frac{d^{2}}{4}+dd_{4}+d_{4}^{2}+h_{4}^{2}-l^{2}+P_{4z}^{2} \end{array}$$

The Equation (15) is widely applied in the kinematics of robots and mechanisms. The equation might be easily solved using trigonometric functions expressed with the tangent of a half angle. Assuming that:

$$t_{i}=\tan\frac{q_{i}}{2}$$

then:

$$\sin q_{i}=\frac{2t_{i}}{1+t_{i}^{2}}\;\;\;\;\;\;\;\;\;\;\;\cos q_{i}=\frac{1-t_{i}^{2}}{1+t_{i}^{2}}$$

By using the above dependencies in the Equation (15) and transforming them appropriately in relation to *t_i_*, a quadratic equation in the following form is achieved:

(20) $$\left(G_{i}-F_{i}\right)t_{i}^{2}+2E_{i}t_{i}+F_{i}+G_{i}=0\;\left(i=1,\;2,\;3,\;4\right)$$

whose distinguishing feature Δ*_i_* equals:

(21) $$\Delta_{i}=4\left(E_{i}^{2}+F_{i}^{2}-G_{i}^{2}\right)\;\left(i=1,\;2,\;3,\;4\right)$$

The Equation (20) has two solutions:

(22) $$t_{i1,2}=\frac{-E_{i}\pm \sqrt{E_{i}^{2}+F_{i}^{2}-G_{i}^{2}}}{G_{i}-F_{i}}\;\left(i=1,\;2,\;3,\;4\right)$$

**Figure 7:**
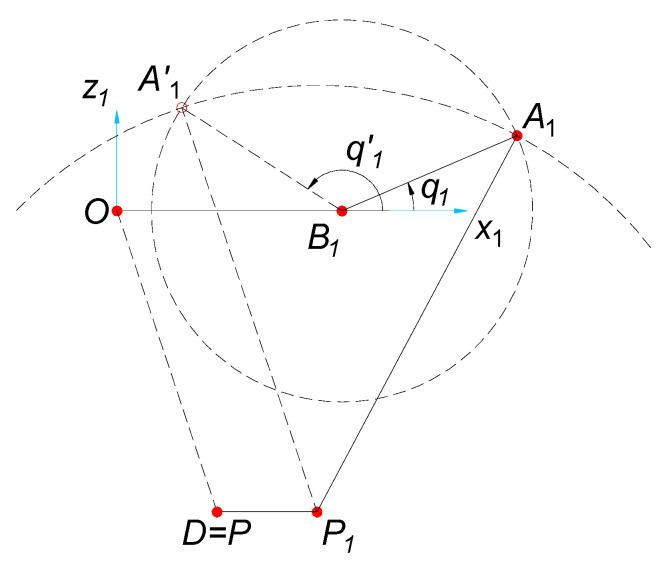
Possible solutions of the problem of inverse kinematic for *delta* 4-DoF parallel manipulator.

The geometric analysis of the manipulator allows to eliminate one out of two solutions for each system Equation (15)—Figure 7. Eventually, the sought value of the configuration coordinate *q_i_* (which is the solution of the problem of the inverse kinematic) for each robot arm under consideration equals:

(23) $$q_{i}=2\tan^{-1}\left(\frac{-E_{i}-\sqrt{E_{i}^{2}+F_{i}^{2}-G_{i}^{2}}}{G_{i}-F_{i}}\right)\;i=\left(1,\;2,\;3,\;4\right)$$

Table 1 includes all geometric parameters of the robot, their real dimensions that result from the construction of the designed manipulator.

### 3.3. Manipulator Operating Space

Manipulator operating space is a set of points in space to which its end might be led. Both size and shape of the operating space depends on the type of the robot mechanical construction, geometric dimensions, and the range of movement of particular manipulator elements.

**Figure 8:**
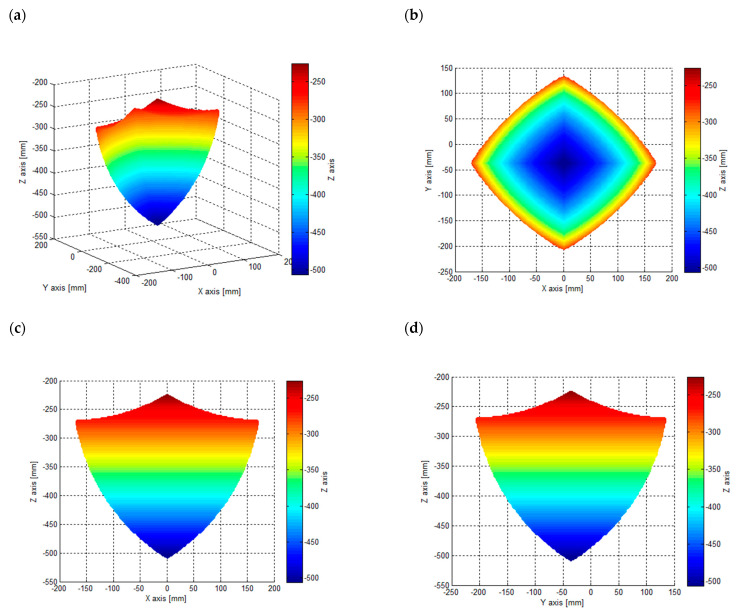
Manipulator operating space: (**a**) general overview, (**b**) view in XY plane, (**c**) view in XZ plane, (**d**) view in YZ plane.

For the designed *delta* 4-DoF manipulator, the operating space was determined with the use of space discretization method for a set of points. Therefore, a net of equidistant (in every axis) points in the form of a cube with the following dimensions x∈〈−170,170〉 mm, y∈〈−210,140〉 mm, z∈〈−510,−220〉 mm was generated. Subsequently, a set of points was chosen for which it was possible to solve the problem of inverse kinematics (under the condition that the articulated variables of all active manipulator arms fulfil the following condition $q_{i}\in \langle -45^{{}^{\circ}},45^{{}^{\circ}}\rangle$). The operating space was determined for a rotary angle of the articulated moveable platform *θ* = 0°. Its overview is presented in Figure 8.

## 4. Design of the Control System of Delta 4-Dof Manipulator

### 4.1. Robot Control System

**Figure 9:**
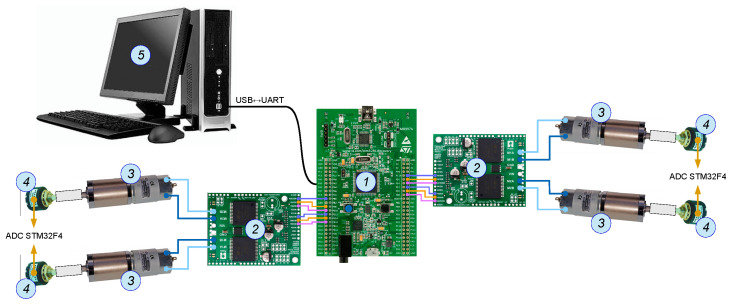
The diagram of control architecture.

The diagram of the control system of the designed *delta* 4-DoF manipulator is presented in Figure 9.

STM32F4DISCOVERY developing platform (1) based on microcontroller STM32F407VGT6 was chosen for control and communication. A ready-made set was taken into consideration, as it is completely sufficient to fulfil the assumed tasks related to control the robot operation. The algorithm of robot control was prepared in Matlab/Simulink with Waijung Blockset toolbox. This is a library dedicated to systems with microcontrollers that originate from STM32F3/STM32F4. Toolbox Waijung Blockset might successfully be applied in rapid prototyping of control systems using runtime sets. Programming DISCOVERY plate consists of generating the source programme code directly from the graphical model of the object built in Simulink, and subsequently, uploading it to the microcontrol memory and testing its performance correctness. The manipulator drives are controlled with the use of VNH5019 (2) controller. It is a bridge H dedicated for motors of direct current powered with a voltage of 5.5 ÷ 24 V with a constant power consumption up to 12 A (temporarily up to 30 A).

Four motors of direct current PG45775243000-264k (3) with a planetary gearbox were applied to drive the robot arms. Motors allow to achieve a rotary moment up to 4.9 Nm with a voltage of 24 V. A precise single-turn linear potentiometer MUP1100 R = 10 K (4), which is characterized by good resolution (10 kΩ) and high linearity tolerance 1%, was applied to measure the angular position of every active arm of the device.

### 4.2. Structure of Robot Control System

**Figure 10:**
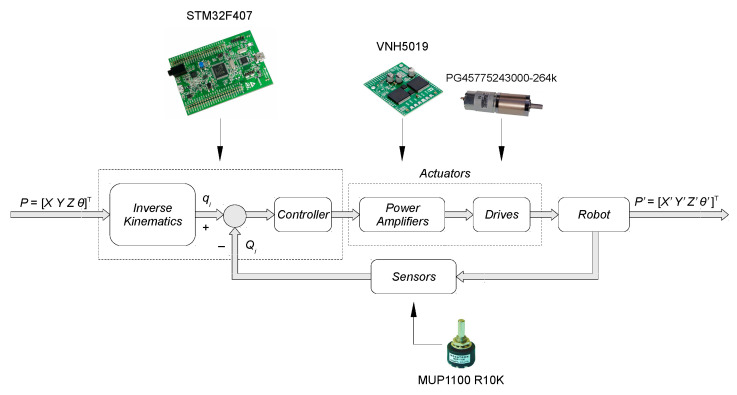
A control diagram of the control system of the designed robot (in the connector space).

So that the designed robot could transport the end-effector to an assigned position in the operating space (Cartesian), it is necessary to control four drives with feedback to the angular position of particular arms. Therefore, a control system was determined whose flow chart in the connector space is presented in Figure 10. On the basis of vector *P* describing the set location (*X*, *Y*, *Z*) and the orientation *θ* of the manipulator operating end in the Cartesian space, the problem of inverse kinematics is solved and corresponding vector of set configurational variables *q_i_* (*i* = 1, 2, 3, 4) of rotary connections of active robot arms is calculated. Vector *q_i_* is an input signal for closed-loop control system with a negative feedback (in the connector space). The control signal generated by the controller is transformed by the executive elements—power amplifiers and the drives controlled by them (motors with gearboxes)—into a signal adapted to control the object (a single manipulator arm).

The measuring element converts the measured physical quantity into voltage which is converted to the adjustable volume *Q_i_* (the angular position of a single arm is measured) through the ADC transmitter of STM32F4 system and appropriate scaling. Subsequently, this quantity is implemented directly to the input of the controller through the accumulated node (differential circuit).

### 4.3. Identification of the Mathematical Model of DC Motor with a Gearbox

**Figure 11:**
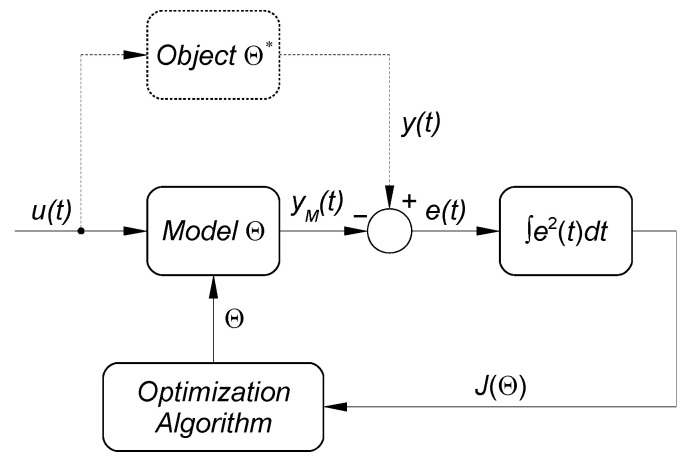
A diagram of the process of object identification. *u*(*t*)—input signal; *y*(*t*)—object response; *y_M_*(*t*)—response of the object model; *e*(*t*) = *y*(*t*) − *y_M_*(*t*)—identification error; $J\left(\Theta \right)=\int_{0}^{T}e^{2}\left(t\right)dt$—quality indicator of compatibility of model and object; Θ *i* Θ*—respectively, model and real object parameters.

The values of coefficients of the model describing DC motor with a gearbox were achieved as a result of identification that was conducted with the use of the so called *output-error* method. The flow chart thereof is presented in Figure 11.

For the purpose of this experiment, the measurement of the input *u*(*t*)and output signal *y*(*t*) of the object was conducted. The input signal was the voltage with the scope of –12 ÷ 12 V, but the response of the object was the angular position (in degrees) of the output shaft of the motor gearbox. The mathematical model of the object was assumed in the form of a transfer function of the inertial element of the second row:

(24) $$G\left(s\right)=\frac{K}{s^{2}+T_{1}s+T_{2}}$$

In order to determine the sought model parameters, a proper script in Matlab was written. After calling this script, the following coefficients of the DC motor with gearbox were achieved:

*K* = 9.4138      *T*_1_ = 3.5865 [s]      *T*_2_ = 0.1790 [s]

After substituting those values to (24), the mathematical model of the motor with a gearbox took the following form:

(25) $$G\left(s\right)=\frac{9.414}{s^{2}+3.586s+0.179}$$

**Figure 12:**
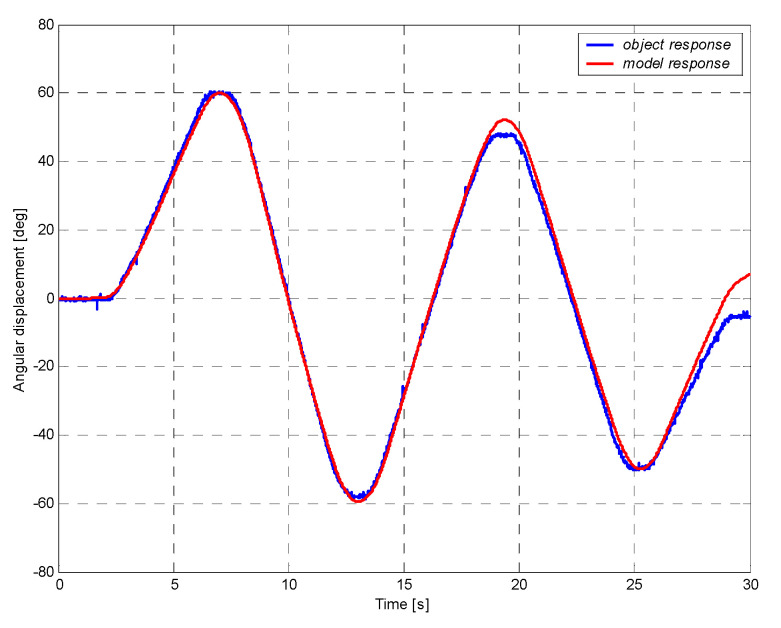
Comparison of the response of model and real object of DC motor with a gearbox.

The model was verified by comparing its responses with the response of the real object (Figure 12). The obtained course of model response for a given input signal is similar to the response of the object, which indicates that the conducted identification process might be considered as correct.

### 4.4. Choice of Type and Settings of the Controller

#### 4.4.1. Choice of Settings of the Controller with the Use of Self-Tuning Method in Matlab/Simulink

**Figure 13:**
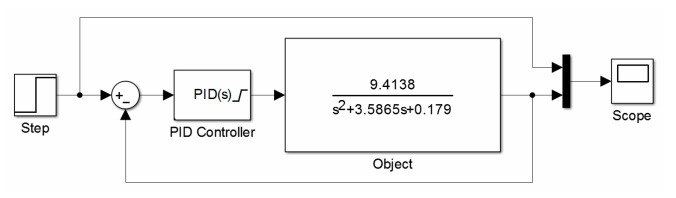
Model of a closed-loop control system of PID controller with auto-tuning.

The control of the angular position of the various arms of the designed active manipulator was based on the PID control. Matlab software and its composite package Simulink were used in order to choose the settings of the controller. For this purpose, a model of a closed-loop control system (as shown in Figure 13), using PID controller with auto-tuning function and designated transmittance of the model of DC motor with a gearbox, was built.

**Figure 14:**
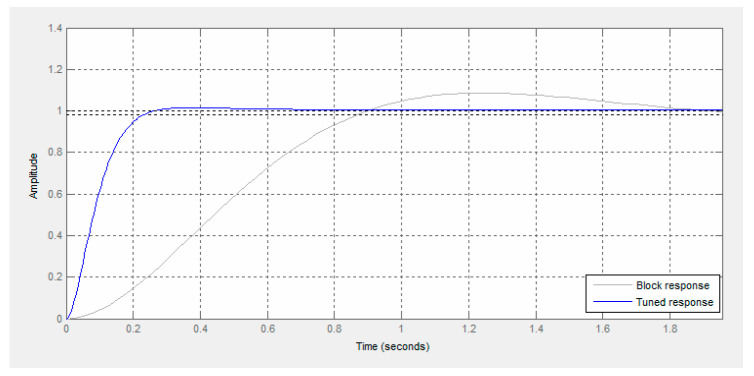
Fluctuating response of the object for chosen PID controller settings.

Selected settings of the controller for an object described with an Equation (25) are shown in Table 2. PID control described with the following relation (26) was used in the simulation tests of the manipulator control system (Figure 14):

(26) $$G_{R}\left(s\right)=kp\left(1+\frac{1}{T_{i}s}+T_{d}s\right)$$

#### 4.4.2. Choice of Settings of the Controller with the Method Based on the Approximation of the Parameters of the Fluctuating Response

**Figure 15:**
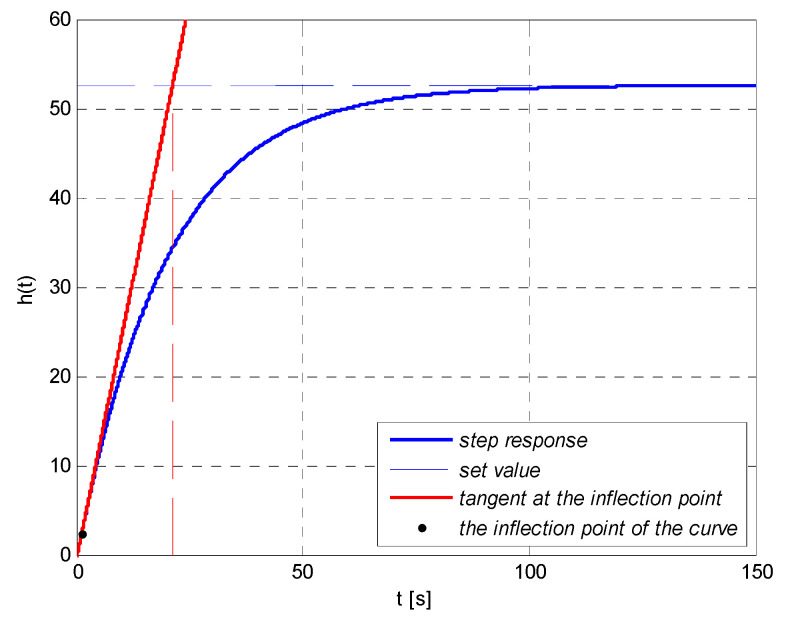
The approximation of the parameters of the fluctuating response of an object described with the following dependency (25).

In order to compare the quality of the adjustment of the control system with the controller settings chosen with the use of auto-tuning method, settings of PID controller with the use of the method based on the approximation of the parameters of the fluctuating response were determined. For this purpose, Matlab environment was exploited, in which an application which allows fulfilling this task was prepared. The results—graphic interpretation, parameters of the fluctuating response, and controller settings—are shown in Figure 15 and Table 3.

### 4.5. Simulation Research of the Manipulator Control System

**Figure 16:**
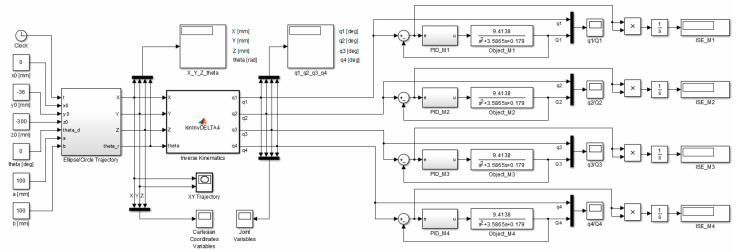
Simulation diagram of the manipulator control system in Matlab/Simulink.

In order to select the optimal PID controller settings which provide good quality of control, a simulation study of the control system was conducted. Figure 16 shows the model built in Matlab/Simulink.

**Figure 17:**
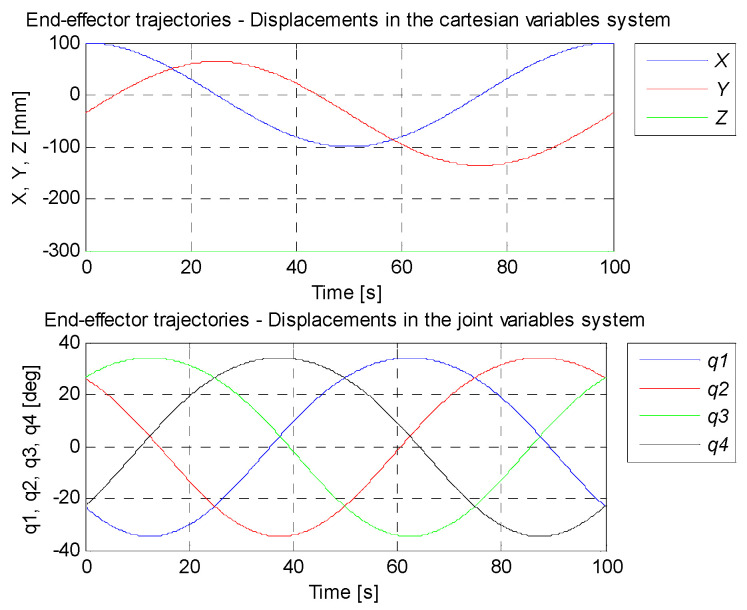
The course of Cartesian (**top**) and configurational (**bottom**) variables for the given track.

**Figure 18:**
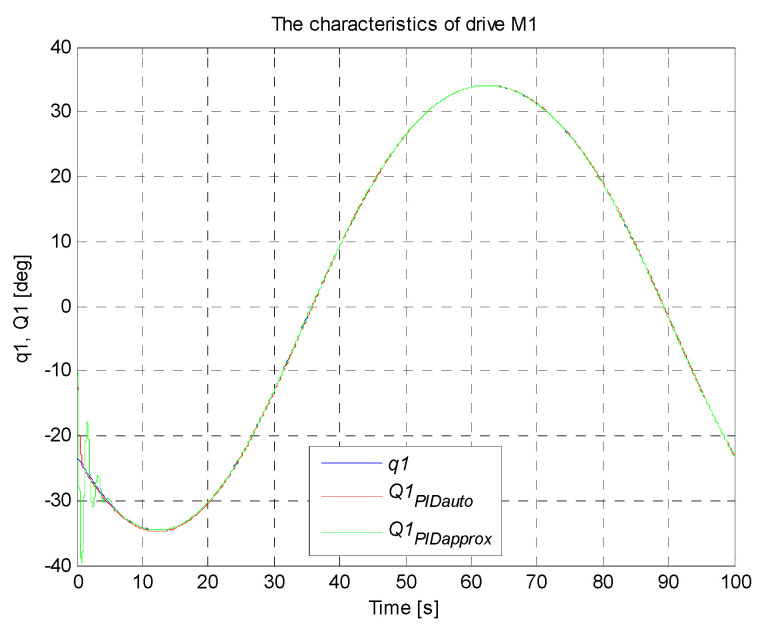
The courses of set and controlled configurational variables for the drive of the first arm.

**Figure 19:**
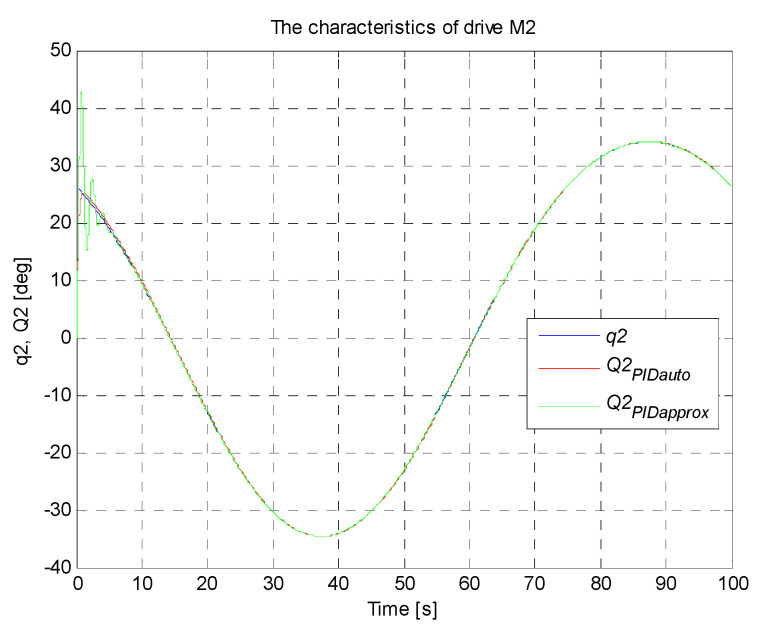
The courses of set and controlled configurational variables for the drive of the second arm.

**Figure 20:**
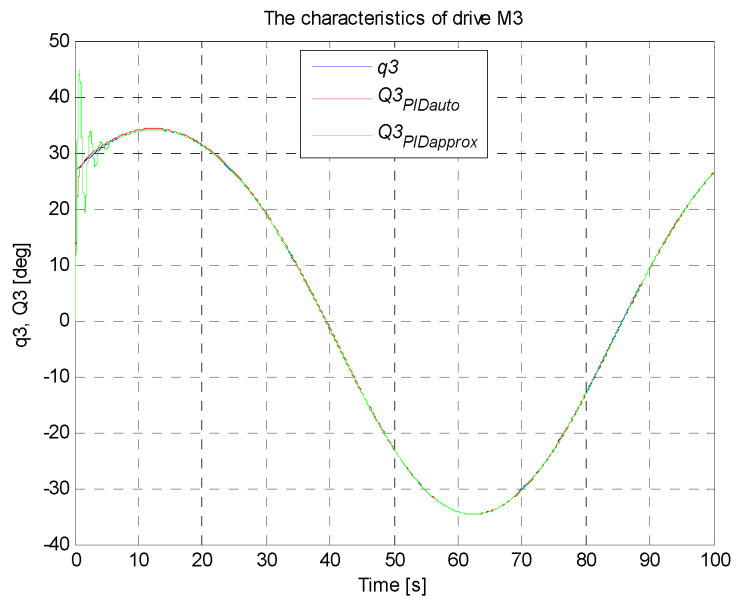
The courses of set and controlled configurational variables for the drive of the third arm.

**Figure 21:**
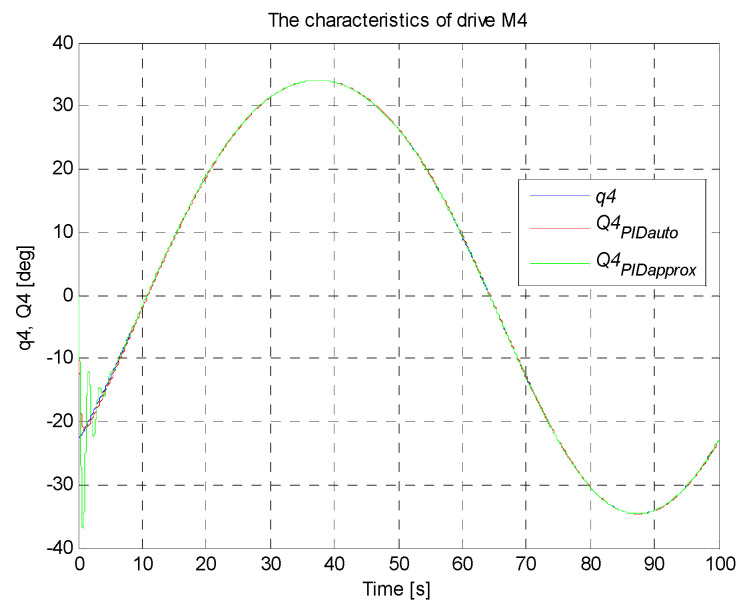
The courses of set and controlled configurational variables for the drive of the fourth arm.

A circle with its center in the point (0,−36,−300) mm and a radius of 100 mm constitutes the set trajectory within the scope of tasks of the robot. The movable platform of the robot moves with a constant angular orientation of 0°. On the basis of the control deviation, an indicator (criterion) of the control quality in the form of ISE integral (*Integral Squared Error*) was calculated. Simulation time was 100 s with a constant sampling time of 0.01 s. The simulation results for the trajectory and the PID controller settings that were determined, are illustrated in Figure 17, Figure 18, Figure 19, Figure 20 and Figure 21. Table 4 presents the designated values of the index of the control quality—ISE integral.

In order to check the operation of the manipulator, it was proposed to map the trajectory of the platform motion in the shape of a circle with a radius of 100 mm in Cartesian coordinates. For the designed circular trajectory, tests were carried out on the proposed robot drives in articulated coordinates, which are presented in Figure 17, Figure 18, Figure 19, Figure 20 and Figure 21.

The best control quality was obtained for the control system with PID controller settings that were chosen with the use auto-tuning method in Matlab/Simulink. The courses of set and controlled quantities in the connector space for every robot arm coincide with good accuracy. The obtained values of ISE index are smaller than those obtained for the system of controller settings chosen on the basis of the method of the fluctuating response. The control quality for the system with PID controller settings that were chosen with the use of the second method is noticeably worse, which is confirmed by a fairly large value of ISE integral (in the initial simulation phase, visible significant oscillations of the controlled quantity around the set point, followed by stabilization). The studies also confirmed that the PID controller with settings precisely matched fulfils the task of the control system well.

### 4.6. Structures of the Complied Programmes on the Runtime Set STM32F4 Discovery

In order to accomplish the test applications and rapid prototyping of the control algorithm that were described below, the development platform STM32F4 was chosen. These programmes were built in the form of flow charts in Matlab/Simulink 2013a with Waijung Blockset (version 15.04) library installed therein. The applied blocks were executed with the same time resolution (parameter *Sample time*) that equals 0.01 s.

#### 4.6.1. Programme Target_DELTA4.slx

**Figure 22:**
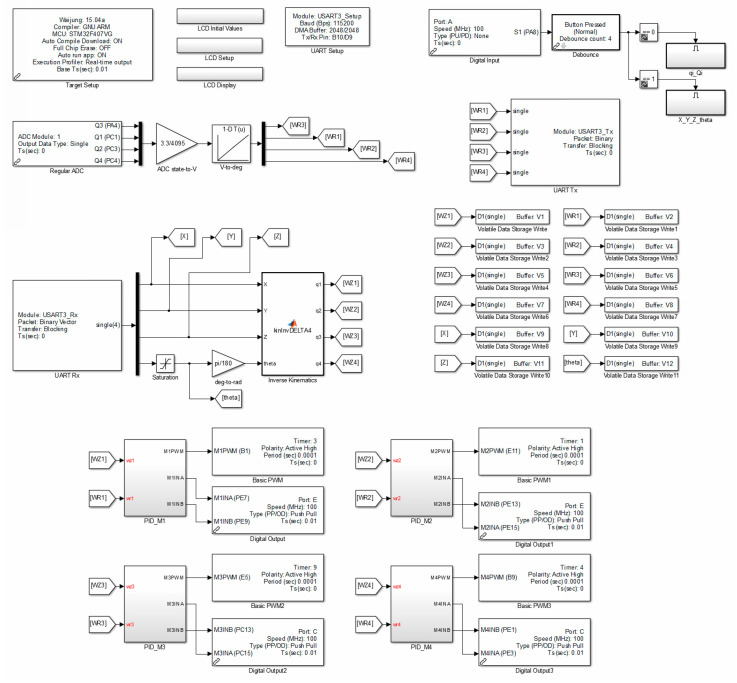
A flow chart of the algorithm of position and orientation control of the operating element of *delta* 4-DoF manipulator (Matlab/Simulink and Waijung Blockset).

Figure 22 shows the flow chart of the structure of the main algorithm of the manipulator drive control.

This algorithm is based on a closed-loop control system with the feedback out of the angular position of particular robot arms (the structure thereof is provided and described in Section 4.2). The developed programme allows to control the position and orientation of the operating end of the manipulator on the basis of the Cartesian coordinates of successive points of the track and the values of the rotary angle of the end-effector, which the robot has to develop, that are transferred from the computer (through USB↔UART converter). On the basis of these values, inverted kinematics are recalculated on a regular basis and new coordinate values of the articulated drives of the active manipulator elements (rotary angle, in degrees) are calculated. The deviation signal is a difference of the set quantity (the value of a configuration variable calculated through the control system) and the controlled quantity (the measured value of the generated rotary angle of the arm) of the angular position of the active manipulator elements. Subsequently, this difference is processed by the PID control. The controller generates a control signal in the form of PWM (the range of the filling −100% to 100%) and the voltage polarization of the power amplifier that controls the DC motor (control of speed and direction of rotation).

The application also enables operation of the LCD display, which presents the values of the articulated *q_i_* and adjustable variable *Q_i_*, point coordinates (*X*, *Y*, *Z*), and orientation *θ* of the operating end.

#### 4.6.2. Host_DELTA4.slx Programme

**Figure 23:**
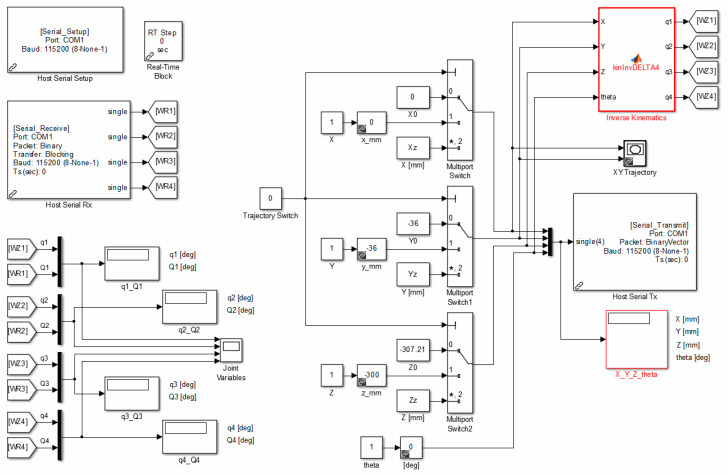
A flow chart of Host_DELTA4.slx in Matlab/Simulink.

Figure 23 shows Host_DELTA4.slx programme. It is used for serial communication (data transmission and reception) with the runtime set STM32F4, where the algorithm of robot control is implemented.

The application of the *Real-Time Block* means that the simulation is performed within near-real-time. During the operation of the programme, numerical values (blocks *Display*) of the position and orientation of the operating element, both set and controlled configuration variables are presented on a regular basis, and time courses (block *Scope*)—track visualization on a *XY* plane, courses of set quantities and quantities produced by the drives in connectors space are generated as well.

## 5. Conclusions

In this article, the reference to the existing structures of delta-4 DoF robots was made. The components of the developed manipulator construction were presented. The analytical solution of the problem of inverse kinematics was formulated and derived. The concept of the developed control system (in the connector space) and a list of the selected components was presented. For the control object of the DC motor with a gearbox, the parameters of the model in the form of a transfer function were determined with the use of identification of method of the output error. The type and controller settings were selected on the basis of two methods. Simulation studies of the manipulator control system for PID controller were conducted. The device control algorithm was implemented with the microprocessor runtime set STM32F4. The developed program allows control of the position and orientation of the operating end of the manipulator on the basis of the Cartesian coordinates and the values of the rotary angle of the end-effector which the robot has to develop, that are transferred from the computer (through USB↔UART converter). Additionally, the application allows operation of the LCD display. Generally available inexpensive runtime sets with microprocessors that originate from the family of STM32F4 allow both rapid prototyping of complex control algorithms and solving kinematics equations as well as their hardware implementation. Such an approach while designing robots allows rapid development and control of complex kinematic structures not only of parallel robots but also serial and hybrid structures.

## Figures and Tables

**Table 1:** The most important geometric parameters of the designed manipulator.

Marking	Description	Value (mm)
*R*	Distance between the beginning of the reference system *O* and the driven axis of the active arm	185
*L*	Length of the active arm	386
*l*	Length of the passive arm	200
*d*	Length of the main element of the moveable platform (along the axis *x*)	102
*d_i_* (*i* = 1, 2, 3, 4)	Distance between points *C_i_* and *P_i_* of the moveable platform (along the axis *x*)	9
*h*	Length of the connectors of the articulated quadrangle of the moveable platform	72
*h_i_* (*i* = 1, 2, 3, 4)	Distance between points *C_i_* and *P_i_* of the moveable platform (along the axis *y*)	24

**Table 2:** PID regulator settings chosen with the auto-tuning method in Matlab/Simulink.

PID Regulator Settings	
*P_auto_*	4.36210
*I_auto_*	0.38231
*D_auto_*	0.99919
*N_auto_*	37.2929

**Table 3:** The determined parameters of the fluctuating response of the object (25) and calculated PID controller settings.

The Pareamters of the Object	
Object Proportionality Factor *k*	52.5561
Delay *T*_0_ [s]	0.2445
Time constant *T* [s]	20.9963
PID controller settings	
*k_p_*	2.2880
*T_i_* [s]	0.3178
*T_d_* [s]	0.1222

**Table 4:** ISE indicators for simulation studies of the control system.

PID Controller Settings	ISE Indicator			
	Drive I	Drive II	Drive III	Drive IV
PID_auto_	91.45	124	126	88.85
PID_approx_	234.8	305.4	307.9	231.7

## Data Availability

Not applicable.
